# Causes and characteristics of death in patients with acute hypoxemic respiratory failure and acute respiratory distress syndrome: a retrospective cohort study

**DOI:** 10.1186/s13054-020-03108-w

**Published:** 2020-07-03

**Authors:** Scott W. Ketcham, Yub Raj Sedhai, H. Catherine Miller, Thomas C. Bolig, Amy Ludwig, Ivan Co, Dru Claar, Jakob I. McSparron, Hallie C. Prescott, Michael W. Sjoding

**Affiliations:** 1 Department of Internal Medicine, University of Michigan, 1500 East Medical Center Drive, Taubman Center, Ann Arbor, MI 48109 USA; 2 Department of Emergency Medicine, University of Michigan, Ann Arbor, MI USA; 3 Institute for Healthcare Policy and Innovation, Division of Pulmonary and Critical Care Medicine, Department of Internal Medicine, University of Michigan, Ann Arbor, MI USA; 4 VA Center for Clinical Management Research, Ann Arbor, MI USA

**Keywords:** Acute respiratory distress syndrome, Acute hypoxemic respiratory failure, Mortality, Cause of death

## Abstract

**Background:**

Acute hypoxemic respiratory failure (AHRF) and acute respiratory distress syndrome (ARDS) are associated with high in-hospital mortality. However, in cohorts of ARDS patients from the 1990s, patients more commonly died from sepsis or multi-organ failure rather than refractory hypoxemia. Given increased attention to lung-protective ventilation and sepsis treatment in the past 25 years, we hypothesized that causes of death may be different among contemporary cohorts. These differences may provide clinicians with insight into targets for future therapeutic interventions.

**Methods:**

We identified adult patients hospitalized at a single tertiary care center (2016–2017) with AHRF, defined as PaO_2_/FiO_2_ ≤ 300 while receiving invasive mechanical ventilation for > 12 h, who died during hospitalization. ARDS was adjudicated by multiple physicians using the Berlin definition. Separate abstractors blinded to ARDS status collected data on organ dysfunction and withdrawal of life support using a standardized tool. The primary cause of death was defined as the organ system that most directly contributed to death or withdrawal of life support.

**Results:**

We identified 385 decedents with AHRF, of whom 127 (33%) had ARDS. The most common primary causes of death were sepsis (26%), pulmonary dysfunction (22%), and neurologic dysfunction (19%). Multi-organ failure was present in 70% at time of death, most commonly due to sepsis (50% of all patients), and 70% were on significant respiratory support at the time of death. Only 2% of patients had insupportable oxygenation or ventilation. Eighty-five percent died following withdrawal of life support. Patients with ARDS more often had pulmonary dysfunction as the primary cause of death (28% vs 19%; *p* = 0.04) and were also more likely to die while requiring significant respiratory support (82% vs 64%; *p* <  0.01).

**Conclusions:**

In this contemporary cohort of patients with AHRF, the most common primary causes of death were sepsis and pulmonary dysfunction, but few patients had insupportable oxygenation or ventilation. The vast majority of deaths occurred after withdrawal of life support. ARDS patients were more likely to have pulmonary dysfunction as the primary cause of death and die while requiring significant respiratory support compared to patients without ARDS.

## Background

Acute hypoxemic respiratory failure (AHRF) is among the most common causes of critical illness, with a hospital mortality of approximately 30% [1]. In patients meeting the definition of acute respiratory distress syndrome (ARDS), mortality is approximately 40% [2]. However, while AHRF and ARDS are each defined by severe hypoxemia and associated with high mortality, death due to refractory hypoxemia is reportedly rare. In cohorts of ARDS patients treated in the 1990s, only 13–19% of deaths were due to refractory hypoxemia, while deaths due to multi-organ failure from sepsis were the cause of up to 50% of deaths [3]. These findings suggested that therapies focused on reducing the complications of sepsis would have a greater impact at improving ARDS survival than therapies for severe hypoxia.

Since the 1990s, however, cause of death specifically related to organ system dysfunction has not been described despite substantial evolution in critical care practices. Ventilator management now focuses on minimizing ventilator-induced lung injury, as opposed to normalizing oxygenation and ventilation [4], which may have led to further reduction in death due to refractory hypoxemia. In addition, there has been growing attention to minimization of sedation, early mobilization, and sepsis recognition and treatment, the latter of which may mitigate mortality due to sepsis [5–8]. Finally, there has been an increased focus on palliative care in the intensive care unit (ICU), which may lead to earlier treatment limitations [9–11]. Because of these changes in practice and how they may affect causes of death in the ICU, we hypothesized that causes of death among AHRF and ARDS patients may be different from historical cohorts. An updated understanding of the causes of death in these populations would help identify the most important targets for new therapies and help direct future investigation to improve survival. We sought to determine the causes and circumstances of death in a contemporary cohort of AHRF patients, and assess whether causes of death differed among patients with and without ARDS.

## Methods

### Cohort

We performed a retrospective cohort study of adult patients (aged ≥ 18 years) hospitalized at Michigan Medicine (January 1, 2016, to December 30, 2017) with AHRF who experienced in-hospital death. Patients were identified via an electronic query tool of the electronic health record. As in prior studies [12, 13], patients were defined as having AHRF when the following criteria were met: (1) receipt of invasive mechanical ventilation for at least 12 h (to exclude routine post-operative ventilation) in the medical, surgical, cardiac, trauma, or neurologic ICU, and (2) a PaO_2_/FIO_2_ ratio ≤ 300. Low-tidal volume ventilation and protocols for daily awakening and spontaneous breathing trials for mechanically ventilated patients were employed [14]. Demographics, comorbidities, highest sequential organ failure assessment (SOFA) score within the first 24 h of AHRF onset, the lowest Glasgow Coma Scale during the 72 h prior to death, and ICU setting were also collected from the electronic health record through use of the electronic query tool.

Patients were classified as having ARDS by multiple physician adjudication as part of a prior study [12]. Specifically, two critical-care trained physicians reviewed each AHRF hospitalization to determine whether patients met Berlin Criteria [15, 16] for ARDS: (1) new or worsening respiratory symptoms began within 1 week of a known clinical insult, (2) PaO_2_/FIO_2_ ≤ 300 while receiving a positive end-expiratory pressure ≥ 5 cm H_2_O, (3) bilateral opacities on chest x-ray, (4) unlikely to be cardiogenic pulmonary edema, and (5) no other explanation for these findings. Disagreement between physicians was resolved by a third physician in 21% of patients [12]. In addition to ARDS status, specific AHRF or ARDS risk factors were collected as part of the prior study (pneumonia, aspiration, non-pulmonary sepsis, non-cardiogenic shock, major trauma, major surgery, transfusion, pancreatitis, major burn, inhalation injury, vasculitis, pulmonary contusion, drowning, or none) [12]. Patients transferred from another hospital were excluded as we were unable to reliably determine ARDS status, AHRF risk factors, or illness severity on presentation.

### Chart abstraction

Patient data were reviewed by one of 5 internal medicine-trained physicians who did not participate in the adjudication of ARDS and were blinded to adjudicated ARDS status. Data regarding causes and circumstances of death were collected using a structured abstraction form (Appendix 1, Online Supplement). Specifically, we abstracted presence and severity of sepsis, presence and severity of organ system dysfunction, withdrawal of life-sustaining treatments, and cause of death, as described further below. All data required for abstractions were available in the electronic medical record. To ensure consistency across reviewers, excellent inter-rater reliability was demonstrated on an initial test set of 10 charts (Appendix 2, Online Supplement).

### Organ system dysfunction

For each patient, we assessed for sepsis and dysfunction of 8 organ systems during the 72 h prior to death. We classified sepsis and each organ dysfunction as severe or irreversible using definitions from a prior study by Stapleton et al. [3], with the following changes (Table 1). We changed the sepsis definition to align with Sepsis-3 (Appendix 3, Online Supplement). In addition, we changed the definition of severe pulmonary dysfunction from specific diagnoses (ARDS, bilobar pneumonia, bronchopleural fistula, or pulmonary embolism) to receipt of significant respiratory support (high-flow oxygen, invasive mechanical ventilation, or non-invasive positive-pressure ventilation) to better capture patients with severe pulmonary dysfunction. If a patient underwent withdrawal of life support before meeting any of the objective organ dysfunction criteria outlined in Table 1, abstractors were instructed to assign irreversible dysfunction to the organ system primarily responsible for the decision to withdraw life support in order to accurately capture cause of death (Appendix 4, Online Supplement). Finally, as in Stapleton et al., we defined multi-organ failure as organ dysfunction in at least two organ systems [3].

**Table 1:** Definitions of sepsis and severe and irreversible organ system dysfunction

System or syndrome	Severe	Irreversible
Sepsis*	Documentation of confirmed or strongly suspected infection and antibiotic use at time of death or within 24 h prior to withdrawal of life support	Septic shock evidenced by documentation of confirmed or strongly suspected infection with mean arterial pressure (MAP) < 65 mmHg^†^ (or) on vasopressors unresponsive to antibiotics (and) no possible surgical intervention. Option was given to apply irreversible dysfunction if care was withdrawn due to poor prognosis related to sepsis.
Pulmonary^°^	Inability to liberate from mechanical ventilation, non-invasive ventilation, or heated high flow nasal cannula due to inadequate oxygenation or ventilation without aforementioned support	Insupportable oxygenation or ventilation defined as PaO_2_ < 40 mmHg on FIO_2_–1.0 for > 2 h or respiratory acidosis with pH < 7.1 on maximum ventilator settings^∞^. Option was given to apply irreversible dysfunction if care was withdrawn due to poor prognosis related to pulmonary organ system dysfunction.
Cardiac	Either cardiac output < 2.0 L/min/m^2^ or documented cardiogenic shock or reversible ventricular fibrillation or asystole	Cardiogenic shock or arrhythmia not responsive to treatment. Option was given to apply irreversible dysfunction if care was withdrawn due to poor prognosis related to cardiac organ system dysfunction.
Neurologic	Glasgow coma scale < 8 for ≥ 3 days	Meets brain death criteria. Option was given to apply irreversible dysfunction if care was withdrawn due to poor prognosis related to neurologic organ system dysfunction.
Hematologic	Microvascular bleeding with either fibrinogen < 100 mg/dL, prothrombin time and partial thromboplastin time > 1.5 times control, or platelets < 60,000/μL	Ongoing microvascular bleeding not surgically correctable with MAP < 65 mmHg not reversible with blood products. Option was given to apply irreversible dysfunction if care was withdrawn due to poor prognosis related to hematologic organ system dysfunction.
Hemorrhage	MAP < 65 mmHg for > 2 h (or requiring vasopressors) necessitating blood transfusions and excluding other causes of hypotension	Uncontrollable “surgical” bleeding from a non-microvascular source. Option was given to apply irreversible dysfunction if care was withdrawn due to poor prognosis related to hemorrhage.
Hepatic	Bilirubin > 5.0 mg/dL and albumin < 2.0 g/dL and prothrombin time or partial thromboplastin time > 1.5 times control	Severe criteria plus hepatic encephalopathy and/or hepatorenal syndrome not responsive to treatment. Option was given to apply irreversible dysfunction if care was withdrawn due to poor prognosis related to hepatic organ system dysfunction.
Gastrointestinal	Resectable ruptured or necrotic bowel, or pancreatitis causing shock (MAP < 65 mmHg for > 2 h or requiring vasopressors)	Inoperable ruptured or necrotic bowel or pancreatitis causing irreversible shock. Option was given to apply irreversible dysfunction if care was withdrawn due to poor prognosis related to gastrointestinal organ system dysfunction.
Renal	Either creatinine > 5.0 mg/dL or requiring hemodialysis	Renal failure with acidosis, hyperkalemia, and/or hypercalcemia causing irreversible cardiac arrest. Option was given to apply irreversible dysfunction if care was withdrawn due to poor prognosis related to renal organ system dysfunction.

*Definition of sepsis changed to reflect current practices. Please see appendix 3, online supplement for previous definition of severe and irreversible sepsis syndrome

^°^Definition of severe pulmonary organ system dysfunction changed to reflect current practices. Previously defined by Stapleton et al. as “[Acute Respiratory Distress Syndrome], bilobar pneumonia, bronchopleural fistula, or pulmonary embolism documented by high-probability ventilation/perfusion scan or pulmonary angiogram”

^∞^*PaO*_*2*_ arterial partial pressure of oxygen, *FIO*_*2*_ fraction of inspired oxygen

^†^Blood pressure parameters previously described by Stapleton et al. as “hypotension” for irreversible hematologic organ system dysfunction or “systolic BP < 80” for severe hemorrhagic and GI organ system dysfunction changed to “MAP < 65 mmHg”

### Cause, features, and circumstances of death

For each patient, we assessed (1) the primary organ system responsible for death, (2) whether death was related to progression of an initial AHRF risk factor or a complication after AHRF, and (3) whether withdrawal of life support occurred prior to death.

The primary organ system responsible for death was defined as the organ dysfunction (Table 1) that most directly resulted in the patient’s death or the decision to withdraw life support (Appendix 5, Online Supplement). For patients with a primary cause of death other than pulmonary dysfunction, cause of death was further classified as being due to progression of an AHRF risk factor (e.g., sepsis, aspiration) or a complication that arose after AHRF onset (Appendix 4, Online Supplement).

Withdrawal of life support was determined from clinical documentation of intent to withdraw life support and/or not escalate life support in the event of clinical decompensation and subsequent removal or non-escalation of life-sustaining interventions.

### Statistical analysis

We present data as numbers (proportions) or medians (inter-quartile range). We compared characteristics of ARDS vs non-ARDS patients using chi-square and Kruskal-Wallis tests and considered *p* <  0.05 to be significant. Data analysis was completed in R. The study was deemed exempt by the institutional review board since all patients were deceased.

## Results

We identified 385 adult patients with AHRF who died during a hospitalization in 2016–2017, of whom 127 (33%) had ARDS. The cohort was a median age of 63 years (55–73), 43% female, 82% white, and had a median SOFA score of 12 (10-14) at AHRF onset. Most patients were admitted to a medical ICU (59%). Patients had a median of 2 (1-3) risk factors for AHRF, most commonly non-cardiogenic shock (59% of patients), transfusion (41%), sepsis (39%), and pneumonia (37%, Table 2).

**Table 2:** Baseline characteristics of AHRF patients

	All patients	ARDS present	ARDS absent	***p***
	***N*** = 385	***N*** = 127	***N*** = 258	
**Baseline features**				
Age (years)—median (IQR)	63 (55–73)	62 (51–71)	64 (56–73)	0.10
Female—no. (%)	164 (43%)	55 (43%)	109 (42%)	0.84
SOFA*—median (IQR)	12 (10–14)	14 (11–17)	12 (10–15)	0.002
Respiration	3 (3–3)	3 (3–4)	3 (3–4)	0.04
Cardiovascular	4 (1–4)	4 (3–4)	4 (1–4)	0.03
Central nervous system	4 (3–4)	4 (3–4)	4 (3–4)	0.56
Liver	0 (0–3)	0 (0–3)	0 (0–3)	0.048
Coagulation	0 (0–3)	0 (0–3)	0 (0–3)	0.048
Renal	2 (1–4)	2 (0.5–4)	2 (1–4)	0.38
**ARDS/AHRF risk factor**—**no. (%)**				
Number of ARDS/AHRF risk factors per patient—median (IQR)	2 (1–3)	3 (2–3)	2 (1–3)	< 0.001
Shock (non-cardiogenic)	227 (59%)	99 (78%)	128 (50%)	< 0.001
Transfusion	158 (41%)	58 (46%)	100 (39%)	0.20
Sepsis (non-pulmonary)	151 (39%)	55 (43%)	96 (37%)	0.30
Pneumonia	143 (37%)	66 (52%)	77 (30%)	< 0.001
Aspiration	58 (15%)	28 (22%)	30 (12%)	0.01
Other^†^	182 (47%)	65 (51%)	117 (45%)	0.33
None	43 (11%)	3 (2%)	40 (16%)	< 0.001
**Intensive care unit setting**—**no. (%)**				
Medical	225 (59%)	92 (72%)	133 (52%)	< 0.001
Cardiac	47 (12%)	4 (3%)	43 (17%)	< 0.001
Surgical	69 (18%)	23 (18%)	46 (18%)	0.95
Trauma/burn	39 (10%)	7 (6%)	32 (12%)	0.04
Neurologic	5 (1%)	1 (1%)	4 (2%)	0.53

*AHRF* acute hypoxemic respiratory failure, *ARDS* acute respiratory distress syndrome

*SOFA sequential organ failure assessment. Represents the highest SOFA score within the first 24 h of AHRF onset

^†^Other risk factors for ARDS/AHRF, each present in < 10% of the cohort, include major trauma (9%), major surgery (7%), pulmonary contusion (3%), pancreatitis (2%), major burn (1%), inhalation injury (1%), vasculitis (< 1%), or drowning (0%)

Patients with ARDS had a higher median SOFA score within the first 24 h of AHRF onset (14 vs 12; *p* = 0.002) and had higher prevalence of pneumonia (52% vs 30%; *p* <  0.001), aspiration (22% vs 12%; *p* = 0.01), and non-cardiogenic shock (78% vs 50%; *p* <  0.001) compared to patients who did not meet the Berlin definition of ARDS (Table 2).

### Organ system dysfunction

Among the 385 patients, there were 1154 occurrences of organ system dysfunction in the 72 h prior to death (eTable 1, Online Supplement). There were 101 (26.2%) patients that had multiple organ systems with irreversible dysfunction. The most common organ system dysfunctions were pulmonary (70%), neurologic (39%), and cardiac (29%). Sepsis was present in 273 (71%) patients and 214 patients (56%) had multi-organ failure prior to death. However, irreversible pulmonary dysfunction was only present in 19 (5%) patients (Table 3)—7 (2% of all patients) with insupportable oxygenation or ventilation, and 12 patients with withdrawal of life support because of a poor pulmonary prognosis.

**Table 3:** Sepsis and organ system dysfunction in the 72 h preceding death

	All patients	ARDS present	ARDS absent	***p***
	***N*** = 385	***N*** = 127	***N*** = 258	
**Sepsis and severe organ system dysfunction—no. (%)**				
Sepsis	273 (71%)	107 (84%)	166 (64%)	< 0.001
Neurologic	151 (39%)	58 (46%)	93 (36%)	0.07
Pulmonary	270 (70%)	104 (82%)	166 (64%)	< 0.001
Cardiac	112 (29%)	31 (24%)	81 (31%)	0.16
Hepatic	33 (9%)	11 (9%)	22 (9%)	0.96
Gastrointestinal	19 (5%)	7 (6%)	12 (5%)	0.71
Hemorrhage	54 (14%)	15 (12%)	39 (15%)	0.38
Hematologic	120 (31%)	52 (41%)	68 (26%)	0.004
Renal	122 (32%)	47 (37%)	75 (29%)	0.12
**Septic shock and irreversible organ system dysfunction—no. (%)**				
Septic shock	136 (35%)	57 (45%)	79 (31%)	0.006
Neurologic	83 (22%)	22 (17%)	61 (24%)	0.16
Pulmonary	19 (5%)	12 (9%)	7 (3%)	0.004
Cardiac	95 (25%)	26 (20%)	69 (27%)	0.18
Hepatic	23 (6%)	8 (6%)	15 (6%)	0.85
Gastrointestinal	18 (5%)	6 (5%)	12 (5%)	0.97
Hemorrhage	33 (9%)	11 (9%)	22 (9%)	0.96
Hematologic	14 (4%)	4 (3%)	10 (4%)	0.72
Renal	10 (3%)	3 (2%)	7 (3%)	0.84
**Multi-organ failure—no. (%)**				
Multi-organ failure	214 (56%)	76 (60%)	138 (53%)	0.24
Sepsis + multi-organ failure	162 (42%)	63 (50%)	99 (38%)	0.04

*ARDS* acute respiratory distress syndrome

Patients with ARDS higher rates of sepsis (84% vs 64%; *p* <  0.001), pulmonary dysfunction (82% vs 64%; *p* <  0.001), irreversible pulmonary dysfunction (9% vs 3%; *p* = 0.004), and hematologic dysfunction (41% vs 26%; *p* = 0.003) compared to patients without ARDS.

### Cause of death

**Fig. 1:**
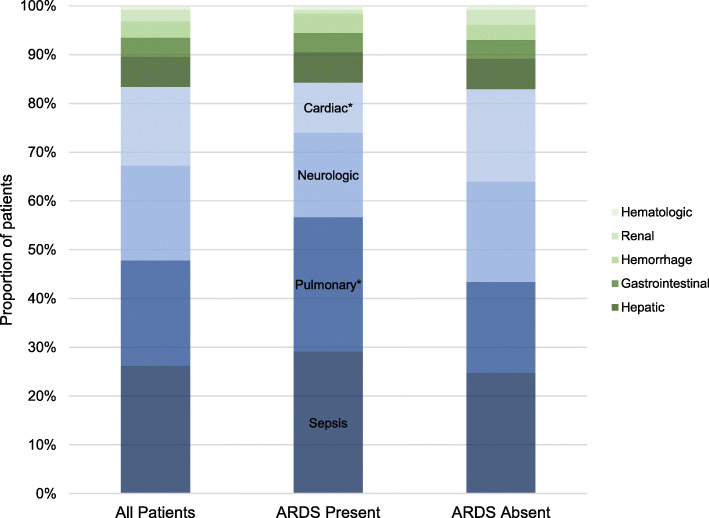
Causes of death among patients with acute hypoxemic respiratory failure. Displays the primary syndrome or organ system dysfunction responsible for death among patients with acute hypoxemic respiratory failure in all patients (*N* = 385) and in patients with acute respiratory distress syndrome (ARDS, *N* = 127) and without ARDS (*N* = 258). *p value < 0.01

Overall, the most common primary causes of death were sepsis (26%), pulmonary dysfunction (22%), and neurologic dysfunction (19%, Fig. 1). Among the 302 patients whose primary cause of death was not pulmonary dysfunction, 212 (55% of all patients) died primarily due to progression of an AHRF risk factor and 90 (23%) died primarily due to complications that arose after the onset of AHRF (Table 4). Cause of death by ICU setting can be found in eTable 2 in the supplementary appendix, with some variation in causes of death noted.

**Table 4:** Characteristics of death in AHRF patients

	All patients	ARDS present	ARDS absent	***p***
	***N*** = 385	***N*** = 127	***N*** = 258	
**Primary syndrome or organ system responsible for death—no. (%)**				
Sepsis	101 (26%)	37 (29%)	64 (25%)	0.36
Pulmonary	83 (22%)	35 (28%)	48 (19%)	0.04
Neurologic	75 (19%)	22 (17%)	53 (20%)	0.45
Cardiac	62 (16%)	13 (10%)	49 (19%)	0.03
Hepatic	24 (6%)	8 (6%)	16 (6%)	0.97
Gastrointestinal	15 (4%)	5 (4%)	10 (4%)	0.98
Hemorrhage	13 (3%)	5 (4%)	8 (3%)	0.67
Renal	9 (2%)	1 (1%)	8 (3%)	0.16
Hematologic	3 (1%)	1 (1%)	2 (1%)	0.99
**Primary cause of death other than pulmonary dysfunction—no. (%)**				
Progression of AHRF risk factor	212 (55%)	57 (45%)	155 (60%)	0.004
Complication arising after AHRF onset	90 (23%)	35 (28%)	55 (21%)	0.17
**Withdrawal of life support—no. (%)**				
Withdrawal of life support	328 (85%)	110 (87%)	218 (84%)	0.58

*AHRF* acute hypoxemic respiratory failure, *ARDS* acute respiratory distress syndrome

ARDS patients were more likely to have a primary cause of death due to pulmonary dysfunction (28% vs 19%; *p* = 0.04) compared to patients without ARDS and less likely to have a primary cause of death from cardiac dysfunction (10% vs 19%; *p* = 0.03, Table 4). In addition, ARDS patients were also more likely to die while receiving substantial respiratory support (82% vs 64%; *p* <  0.001).

The majority of patients (85%) died after withdrawal of life support. The proportion of deaths that occurred after withdrawal of life support did not differ between patients with and without ARDS (87% vs 84%; *p* = 0.58, Table 4).

## Discussion

In this contemporary cohort of 385 adult patients with AHRF, the most common primary causes of death were sepsis, pulmonary dysfunction, and neurologic dysfunction. The majority of patients had multi-organ failure prior to death, most commonly due to sepsis. More than half of patients were receiving substantial respiratory support at the time of death and the vast majority of patients died after withdrawal of life support. Sepsis and pulmonary dysfunction were the top two primary causes of death among both patients with and without ARDS.

Our study is consistent with prior reports indicating that sepsis is the leading cause of death among patients with respiratory failure. Stapleton et al. found that sepsis was the most common cause of death in ARDS patients treated in the 1990s [3]. Despite increased attention to earlier identification and treatment of sepsis in the intervening decades [17, 18], our study found that sepsis remained the most common cause of death in AHRF patients. This is consistent with recent studies showing that sepsis is the leading contributor to death among patients hospitalized for any cause [19]. Sepsis was slightly more common in patients with ARDS than those without ARDS, which may reflect the higher rates of pneumonia and sepsis as risk factors for ARDS. However, it may also suggest that ARDS patients are at a heightened risk for secondary infections compared to patients without ARDS. These findings suggest that therapies targeting sepsis-induced multi-organ dysfunction may have the greatest impact on survival among AHRF patients.

We found only small differences in the causes and circumstances of death among AHRF patients with and without ARDS. Patients with ARDS were more likely to have a pulmonary dysfunction as the primary cause of death and more likely to die while receiving substantial pulmonary support than patients without ARDS. This indicates that the Berlin ARDS definition identifies a subset of patients with AHRF who are more likely to die directly from respiratory failure and would benefit from therapies to enhance resolution of respiratory failure. However, the difference in rates of pulmonary dysfunction as the primary cause of death was relatively small among patients with and without ARDS.

Our study confirms the findings in prior studies indicating that insupportable oxygenation and/or ventilation is rare among patients with respiratory failure. One of the major findings of Stapleton et al.’s study was the relatively low proportion of deaths due to insupportable oxygenation or ventilation, occurring in only 13–19% [3]. Given the increased awareness and effort to treat sepsis in the period after this original study, we hypothesized that pulmonary dysfunction may be a more common primary cause of death in a contemporary AHRF cohort. However, we found that only 22% of patients had pulmonary dysfunction as the primary cause of death, and only a handful of patients (2%) had insupportable oxygenation and/or ventilation. There are several potential explanations for these findings. First, with more consistent use of lung protective ventilation, contemporary AHRF patients may be less likely to develop ventilator induced lung injury and progressive respiratory failure [20]. Second, patients with severe ARDS may be more likely to be initiated on extra-corporeal membrane oxygen therapy prior to developing refractory pulmonary dysfunction [21]. Finally, other strategies such as prone positioning may prevent refractory hypoxemia [22]. However, these hypotheses do not explain why a similar proportion of patients still ultimately die from respiratory failure despite not developing insupportable oxygenation and/or ventilation. While some patients may be supported through the initial phase of their respiratory failure, eventually life support is withdrawn when providers are unable to completely reverse their need for significant respiratory support.

Our study also highlights the increasing proportion of deaths that occur after a decision to withdraw or not escalate life support. Stapleton et al. showed that from 1981 to 1998, the proportion of ARDS deaths that occurred after withdrawal of life support rose from 40 to 67% [3]. Similar trends have been reported for all-cause critically ill patients during this time period [9]. Our study suggests that this trend has continued, as we report that 85% of all deaths among AHRF are now occurring after a decision to withdraw or not escalate life support. Our finding is also consistent with a recent study showing that 90% of deaths among critically ill patients treated in Europe from 2015 to 2016 occurred in the setting of treatment limitations [23].

There are likely several explanations for why a growing proportion of deaths occur after withdraw of life support. Stapleton et al. hypothesized that ICU clinicians have earlier and more frequent goals-of-care discussions [3], as is recommended in various clinical practice guidelines [17]. Indeed, early multidisciplinary meetings with patients and families may lead to an earlier transition to palliative care among patients likely to die [24, 25]. More recently, there has been increased emphasis on family involvement in ICU decision-making and treatment planning, for example, as recommended in the ABCDEF treatment bundle [26]. Overall, the greater emphasis on family involvement in early shared decision making may contribute to earlier transitions to palliation among patients who ultimately die in the ICU [27].

Our study has several limitations. First, as a single-center study, it is possible that it may be lacking generalizability. However, we examined all deaths among patients with AHRF over a 2-year period who were treated in 5 distinct ICUs with different practice patterns. As such, we believe these findings are more broadly applicable. Second, while we tried to harmonize our study definitions to those of Stapleton et al. to facilitate cross-study comparisons, some changes had to be made to account for interval changes in definitions (e.g., sepsis) and treatments (e.g., high-flow oxygen). We limited deviations in study definitions to those deemed absolutely necessary to reflect the current state of ICU practice. Third, patients were classified as having undergone withdrawal of life support regardless of the time lag between withdrawal and death. For patients in whom only minutes elapsed between withdrawal of support and death, death may be more accurately representative of the cessation of medical interventions due to futility. However, our approach for determining rates of withdrawal and the rates of withdrawal we observed are consistent with prior reports [9]. Fourth, given a high rate of withdrawal of life support, the most proximate cause of death is cessation of support. However, our methodology identifies which organ dysfunction or syndrome most directly led to that decision, thereby reflecting the primary pathophysiologic cause of death. Fifth, there may be some subjectivity to assigning cause of death. However, we developed a standardized approach to assess causes of death based on the presence of irreversible and severe organ dysfunctions and confirmed excellent inter-rater reliability in identifying the primary cause of death among reviewers, which serves to strengthen the validity of our methodology. Furthermore, chart review was performed by physicians only, as medical training may limit the subjectivity in identifying cause of death.

## Conclusions

In this contemporary cohort study of 385 patients who died after AHRF, the most common primary causes of death were sepsis and pulmonary dysfunction. Few patients had insupportable oxygenation or ventilation, but most received substantial respiratory support in the 72 h prior to death. The vast majority of deaths occurred after a decision to withdraw or not escalate life support. Patients with ARDS were more likely to have a primary cause of death of pulmonary dysfunction and to receive substantial respiratory support during the 72 h prior to death.

## Supplementary information

**Additional file 1: Appendix 1.** RedCAP Abstraction Tool. **Appendix 2.** Inter-rater Reliability. **Appendix 3.** Previous definition of severe and irreversible sepsis syndrome. **Appendix 4.** Examples. **Appendix 5.** Determining cause of death by organ system. **eTable 1.** Total Organ System Dysfunction. **eTable 2.** Cause of Death by ICU Setting.

## Data Availability

The datasets used and/or analyzed during the current study are available from the corresponding author on reasonable request.

## Abbreviations

AHRF: Acute hypoxemic respiratory failure
ARDS: Acute respiratory distress syndrome
ICU: Intensive care unit
SOFA: Sequential organ failure assessment
