# Supplementary material for: Causes and characteristics of death in patients with acute hypoxemic respiratory failure and acute respiratory distress syndrome: a retrospective cohort study
Source: Crit Care. 2020 Jul 3;24:391. doi: 10.1186/s13054-020-03108-w (PMC7332537; doi:10.1186/s13054-020-03108-w)
Supplement: Supplementary file 1 — Additional file 1: Appendix 1. RedCAP Abstraction Tool. Appendix 2. Inter-rater Reliability. Appendix 3. Previous definition of severe and irreversible sepsis syndrome. Appendix 4. Examples. Appendix 5. Determining cause of death by organ system. eTable 1. Total Organ System Dysfunction. eTable 2. Cause of Death by ICU Setting. [file 13054_2020_3108_MOESM1_ESM.docx]

Causes and Characteristics of Death in Patients with Acute Hypoxemic Respiratory Failure and Acute Respiratory Distress Syndrome: A Retrospective Cohort Study

**Online Supplemental Material**

Scott W. Ketcham, MD; Yub Raj Sedhai, MD; H. Catherine Miller, MD; Thomas C. Bolig, MD; Amy Ludwig, MD; Ivan Co, MD, Dru Claar, MD, Jakob I. McSparron MD, Hallie C. Prescott, MD, MSc; Michael W. Sjoding, MD, MS

**Table of Contents**

Appendix 1: RedCAP Abstraction Tool . . . . . . . . . . . . . . . . . . . . . . . . . . . . . . . . . . . . . . . . . . . . . . . . . . . . . . . . 2

Appendix 2: Inter-rater Reliability . . . . . . . . . . . . . . . . . . . . . . . . . . . . . . . . . . . . . . . . . . . . . . . . . . . . . . . . . . . 6

Appendix 3: Previous definition of severe and irreversible sepsis syndrome . . . . . . . . . . . . . . . . . . . . . . . . 6

Appendix 4: Examples . . . . . . . . . . . . . . . . . . . . . . . . . . . . . . . . . . . . . . . . . . . . . . . . . . . . . . . . . . . . . . . . . . . . . 7

Appendix 5: Determining cause of death by organ system . . . . . . . . . . . . . . . . . . . . . . . . . . . . . . . . . . . . . . . 8

eTable 1: Total Organ System Dysfunction . . . . . . . . . . . . . . . . . . . . . . . . . . . . . . . . . . . . . . . . . . . . . . . . . . . . 9

eTable 2: Cause of Death by ICU Setting . . . . . . . . . . . . . . . . . . . . . . . . . . . . . . . . . . . . . . . . . . . . . . . . . . . . . . 9

**Appendix 1: RedCAP Abstraction Tool**


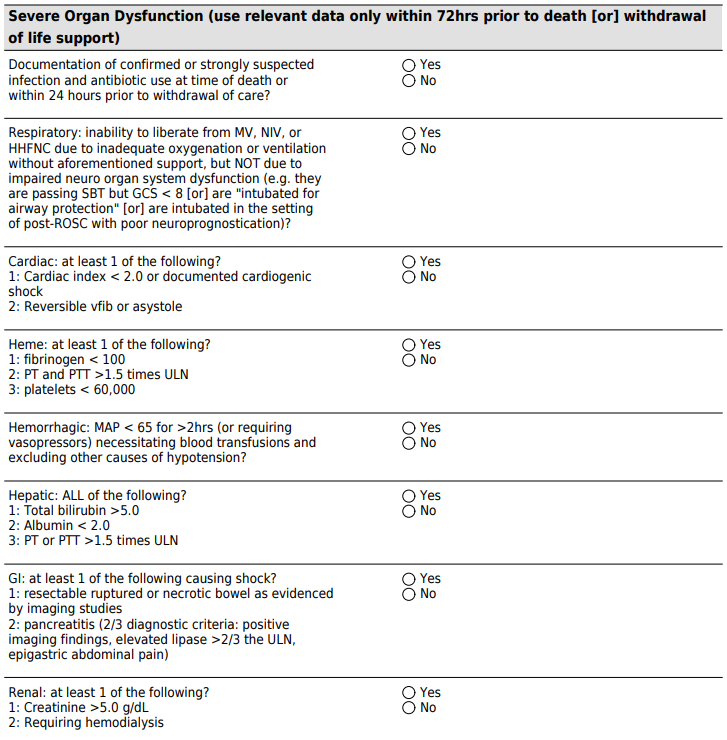


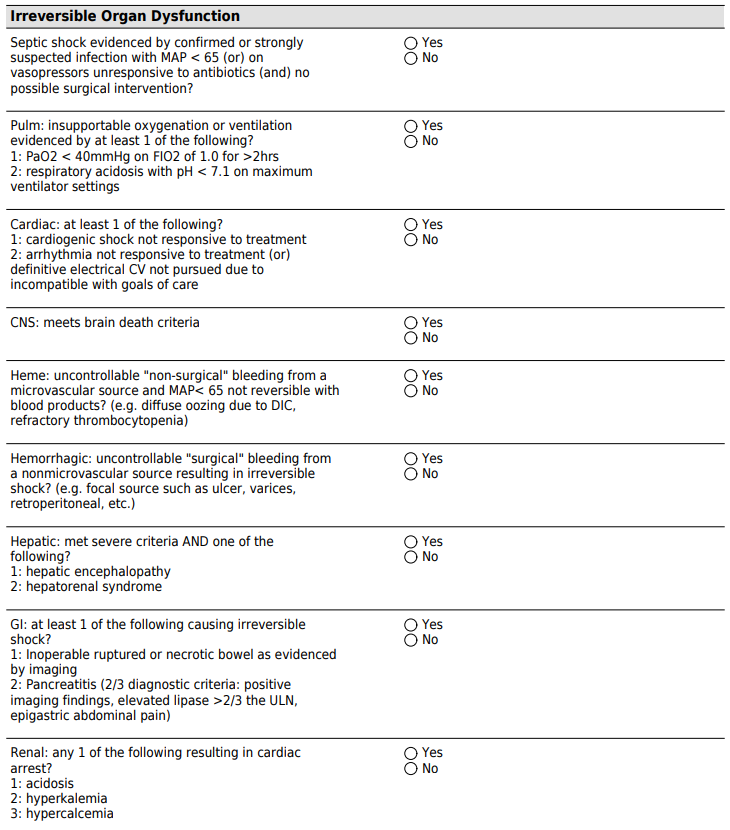


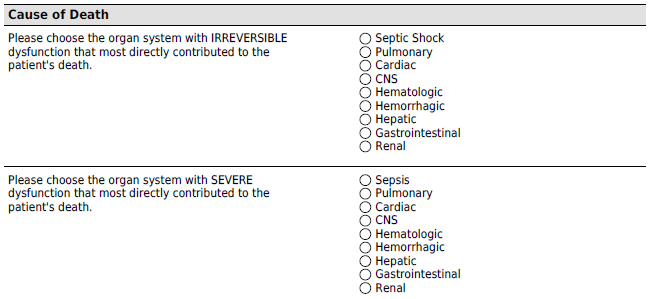


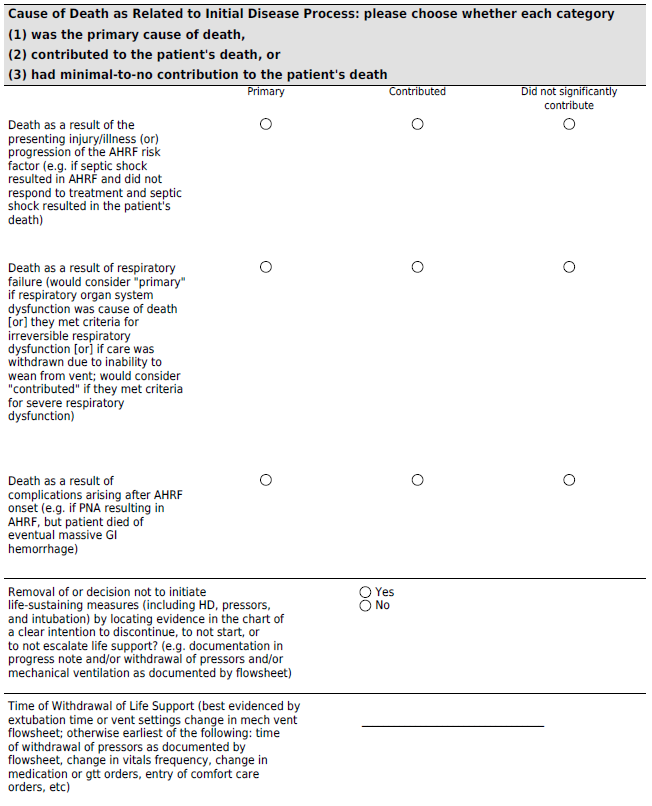


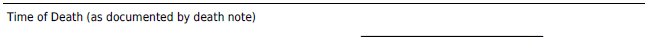


**Appendix 2: Inter-rater Reliability**

In order to measure inter-rater reliability, each of the 5 physician abstractors reviewed the same 10 charts. There was excellent agreement in identifying patients in whom death was primarily the result of progression of the presenting illness (kappa = 0.8217), was primarily the result of pulmonary dysfunction (kappa = 0.9151), or was primarily the result of complications occurring after the onset of respiratory failure (kappa = 0.9008).

**Appendix 3: Previous definition of severe and irreversible sepsis syndrome**(3)

Severe Sepsis Syndrome: At least one criteria from each of the following categories plus one criteria from either category.

Infection/inflammation

1. Temperature < 35°C or ≥ 39°C
2. WBC < 3,000/μL or > 12,000/μL or > 10% bands
3. Positive blood culture of accepted pathogen
4. Known or strongly suspected source of systemic infection with culture known pathogens

Deleterious Systemic Effect

1. Unexplained metabolic acidosis with anion gap > 20
2. Systemic vascular resistance < 800 dyne • s • cm^-5^
3. Unexplained hypotension with systolic BP < 90 for 2 h or receiving vasopressor medications

Irreversible Sepsis Syndrome: severe sepsis syndrome without response to antibiotics and no possible surgical intervention.

**Appendix 4: Examples**

*Irreversible Organ System Dysfunction Assigned due to Withdrawal of Life Support*

For example, suppose a patient was admitted with septic shock as well as AHRF. The patient was only on minimal vasopressor support, but required an FiO2 of 100% and a positive end-expiratory pressure of 15 mmHg to maintain a PaO2 of 45mmHg. Care was subsequently withdrawn due to respiratory failure. While the patient met criteria for irreversible sepsis at the time of death and did not strictly meet criteria for irreversible pulmonary organ system dysfunction, abstractors were encouraged to apply irreversible dysfunction as it most directly related to the reason for withdrawal of care.

*Cause of Death Related to Progression of Presenting AHRF Risk Factor*

For example, if a patient presented with AHRF secondary to extra-pulmonary sepsis and subsequently died of septic shock, then cause of death was attributed to progression of an AHRF risk factor.

*Cause of Death Related to Complications after AHRF Onset*

For example, if a patient presented with AHRF secondary to a pneumonia, but later died of a massive gastrointestinal hemorrhage after a prolonged ICU course, then cause of death was attributed to complications that arose after AHRF onset.

**Appendix 5: Determining cause of death by organ system**

| **eTable 1: Total Organ System Dysfunction** | | |
| --- | --- | --- |
|  | **Severe** | **Irreversible** |
| **Organ system dysfunction – no. (%)** | | |
| Sepsis | 273 (71%) | 136 (35%) |
| Neurologic | 151 (39%) | 83 (22%) |
| Pulmonary | 270 (70%) | 19 (5%) |
| Cardiac | 112 (29%) | 95 (25%) |
| Hepatic | 33 (9%) | 23 (6%) |
| Gastrointestinal | 19 (5%) | 18 (5%) |
| Hemorrhage | 54 (14%) | 33 (9%) |
| Hematologic | 120 (31%) | 14 (4%) |
| Renal | 122 (32%) | 10 (3%) |
| **Total – no.** | **1,154** | **431** |

| **eTable 2: Cause of Death by ICU Setting** | | | | | |
| --- | --- | --- | --- | --- | --- |
|  | **Medical**  **N=225** | **Cardiac**  **N=47** | **Surgical**  **N=69** | **Trauma/Burn**  **N=39** | **Neurologic**  **N=5** |
| **Cause of death – no. (%)** | | | | | |
| Sepsis | 72 (32.0) | 6 (12.8) | 19 (27.5) | 4 (10.3) | 0 (0) |
| Neurologic | 35 (15.6) | 6 (12.8) | 6 (8.7) | 25 (64.1) | 3 (60) |
| Pulmonary | 54 (24.0) | 5 (10.6) | 17 (24.6) | 6 (15.4) | 1 (20) |
| Cardiac | 24 (10.7) | 27 (57.4) | 9 (13.0) | 1 (2.6) | 1 (20) |
| Hepatic | 19 (8.4) | 0 (0) | 5 (7.2) | 0 (0) | 0 (0) |
| Gastrointestinal | 5 (2.2) | 1 (2.1) | 7 (10.1) | 2 (5.1) | 0 (0) |
| Hemorrhage | 8 (3.6) | 1 (2.1) | 4 (5.8) | 0 (0) | 0 (0) |
| Hematologic | 2 (0.9) | 0 (0) | 1 (1.4) | 0 (0) | 0 (0) |
| Renal | 6 (2.7) | 1 (2.1) | 1 (1.4) | 1 (2.6) | 0 (0) |
